# LTCC Strip Electrode Arrays for Gas Electron Multiplier Detectors

**DOI:** 10.3390/s22020623

**Published:** 2022-01-14

**Authors:** Arkadiusz Dąbrowski, Witold Nawrot, Mateusz Czok, Michał Babij, Piotr Bielówka, Karol Malecha

**Affiliations:** 1 Department of Microsystems, Faculty of Electronics, Photonics and Microsystems, Wrocław University of Science and Technology, wyb. S. Wyspiańskiego 27, 50-370 Wrocław, Poland; arkadiusz.dabrowski@pwr.edu.pl (A.D.); witold.nawrot@pwr.edu.pl (W.N.); mateusz.czok@pwr.edu.pl (M.C.); michal.babij@techtra.pl (M.B.); piotr.bielowka@techtra.pl (P.B.); 2 Technology Transfer Agency TECHTRA Sp. z o.o., ul. Duńska 13, 54-427 Wrocław, Poland

**Keywords:** Low Temperature Cofired Ceramics (LTCC), Gas Electron Multiplier (GEM) detector, X-ray, Micro Pattern Gas Detector (MPGD), solar radiation, space sensors

## Abstract

The Low Temperature Cofired Ceramic (LTCC) technology has proven to be highly suitable for 3D microstructures manufacturing in electronic devices due to its excellent electrical and mechanical properties. In this paper, a novel idea of implementing the LTCC structures into high-energy particle detectors technology is proposed. It can be applied in High Energy Physics (HEP) laboratories, where such sophisticated sensors are constantly exposed to particles of the TeV energy range for many years. The most advanced applications of the concept are based on dedicated gas amplifier systems coupled with readout microstructures. Typically, the readout microstructures are made in the Printed Circuit Boards (PCB) technology and processed in a sophisticated and patent-protected way. This article presents the manufacturing process and parameters of the novel microstructures made in the LTCC technology. The structures were implemented into the high-energy particle detector, and the first results are presented.

## 1. Introduction

Introduced in 1968 by Charpak at the European Organization for Nuclear Research (CERN), multiwire proportional chamber revolutionized the particle detection systems in high-energy physics [1]. Over time, new generations of the invention had been implemented for fast detection and localization of charged particles. Solid-state solutions such as silicon, germanium, or scintillation detectors offer the best time and spatial resolution; however, their high costs and limited operation areas are often a drawback, limiting their application in High Energy Physics (HEP) experiments [2].

**Figure 1:**
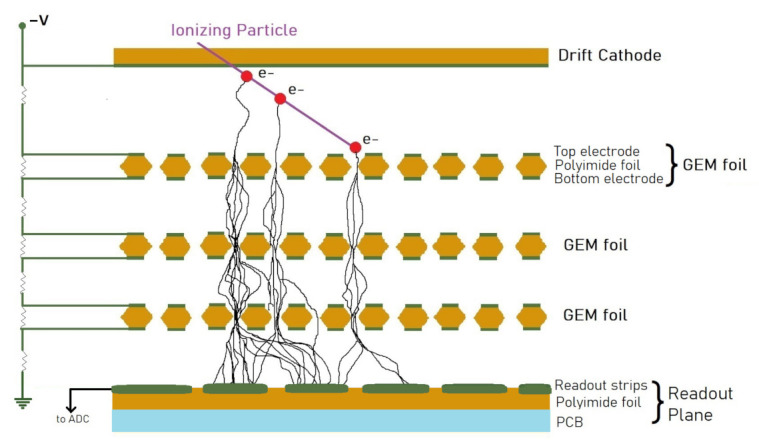
A diagram of a particle detector based on GEM foils.

An alternative for solid-state technology is the gaseous ionization detector technique. It is robust, resistant to high-energy radiation, and last but not least, cost-effective. However, the spatial resolution offered by gaseous technology is lower than that offered by its solid-state counterpart. Over time, that was improved by the implementation of specialized amplifying microstructures. The group of devices that consist of such reliable systems is called the Micro Pattern Gaseous Detectors (MPGD), and the most advanced application, invented at CERN, is the Gas Electron Multipliers (GEM) detector [3]. The principle of GEM detector operation is shown in Figure 1. Ionizing radiation, traversing the thin layer of sensitive gas, releasing electron-ion pairs. Released primary electrons are accelerated in the electric field created between the drift electrode and the top side of the first GEM foil. During the drift through the GEM foil cascade, the electron beam is amplified a few thousand times. The resulting current signal is picked up by a readout plane (typically two layers of perpendicular strips) and measured by a dedicated electronic circuit.

State-of-the-art GEMs are made with 50 μm thick polyimide foil coated on both sides with 5 μm copper layers. The hexagonal pattern of double-conically shaped holes with a diameter of 60–70 μm is typically etched with a pitch of 140 μm into this sandwich-like structure. With suitable potentials applied, it acts as a powerful preamplifier for electrons released by ionizing radiation in the gas, transferring most of the multiplied electron charge to a pickup electrode or another amplifying device [4]. Measured and amplified signals are collected by a readout system. Typically, in the case of GEM, it consists of a matrix of orthogonal conductive lines [5]. The collected charge can be further processed by front-end electronic systems. Such a detector combines the wide dynamic range, good spatial resolution, high gain, and radiation hardness. They have also proved to be robust, light, and offer excellent performance and reliability suited for use in harsh environments.

The goal of the wider project, which this research is a part of, is to design and build a fully operational detector suitable for space applications in the form of a CubeSat module (100 × 100 × 100 mm^3^). Multiple modules, along with necessary power and communication components, could then be formed into a larger system. In this type of mission, the lifetime of a detector is critical, due to the hermetically sealed construction of the gas box. Working gas cannot be exchanged, so outgassing the components reduces gas purity. The application of low outgassing components increases the lifetime of the sealed detector [6]. Morevorer, materials should have as low a coefficient of thermal expansion (CTE) as possible due to the wide range of temperatures occurring during space missions. To meet these unique requirements, a novel readout system was developed. Instead of standard PCB materials, a custom structure made in the Low Temperature Cofired Ceramics (LTCC) is proposed in this research. The LTCC ceramics have significantly lower CTE than FR4 laminates, exhibits very good electrical and mechanical properties, high reliability in harsh environments, good thermal conductivity, and very high operating temperature. Furthermore, it can be brazed, eliminating the need for gluing and resulting outgassing issues. Advanced structuring capabilities and multilayer approach allow for high packaging density, reducing the volume and weight of the device, both of which are very valuable in space missions. This technology is proven through a wide variety of sensors and microsystems [7], including pressure sensors [8], accelerometers [9], magnetic sensors [10], and biosensing microsystems with optical [11] and microwave detection [12]. Due to its robustness and reliability, the LTCC technology is well known in military and space applications [13,14]. The application of ceramic technology in GEM foils had been considered previously [15]. In this work, we focus on the development of the sensing electrode array in the LTCC technology. Such a complete device could be a breakthrough not only in space missions but also in nuclear physics research, such as the NUMEN project, which requires fine resolution but also radiation hardness, to withstand the high rate of impinging particles [16,17].

## 2. Materials and Methods

**Figure 2:**
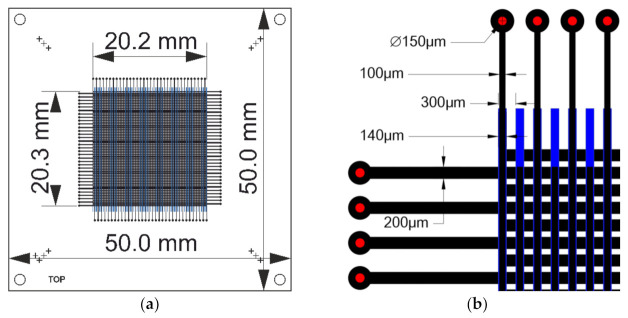
The design of electrode array: (**a**) outer dimensions of active sensing area and substrate; (**b**) dimensions of electrodes and spacing between them.

The schematic of the readout structure is presented in Figure 2. The readout electrode arrays were developed at the Wrocław University of Science and Technology. The design consists of two layers, separated by a dielectric. On each, there are parallel conductive strips that cover an area of 20 × 20 mm^2^. The layers are rotated by 90 degrees, making the lines from each layer orthogonal. The bottom strips were partially obscured by the top ones. Therefore, to maintain a balance between the amount of charge collected by each layer, the widths of the top and bottom lines are different: 100 and 200 μm, respectively. The pitch of the strips remained constant at 300 μm.

**Figure 3:**
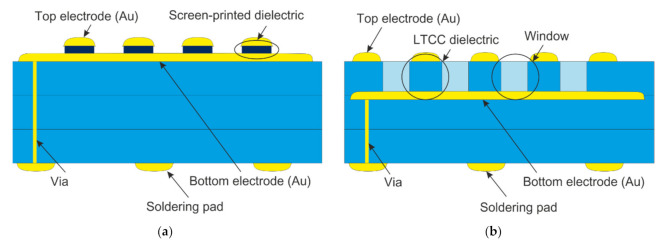
The cross-section schematic for two structural variants of the sensor: (**a**) with screen-printed dielectric layer separating electrodes (Single Substrate, SS); (**b**) with electrodes printed on separate LTCC substrates and stacked together (Multi Substrate, MS).

The readout test structures were manufactured using DuPont 951 Low Temperature Cofired Ceramic (DuPont, Wilmington, DE, USA) green tapes, 254 μm thick. Their shape, basing, and via holes were cut in one step with a ProtoLaser U (LPKF, Garbsen, Germany) Nd:YAG laser, operating at a wavelength of 355 nm. Each structure had been designed to achieve the dimensions of 50 × 50 mm^2^ after the firing process. The vias were filled with the TC0401 Ag/Pd transition paste (Heraeus, Hanau, Germany) by stencil printing and then dried at 100 °C for 10 min. Solder pads were screen printed on the bottom of the substrate, using first the 9145R silver paste (DuPont, USA) and subsequently the 963 silver-palladium paste (Electroscience Laboratory, Columbus, OH, USA). The bottom readout electrodes were then screen-printed with the 5742 gold thick film paste (DuPont, USA). All thick films were deposited using the Aurel VS1520A screen printer (Aurel, Modigliana, Italy) and a 325 mesh steel screen. To provide electrical insulation between the top and bottom conductive layers, a dielectric separation layer was needed. In order to obtain the best results, two different approaches were evaluated (Figure 3). The first approach consisted of multiple screen-printed layers on a single substrate (SS). In the second, each layer of electrodes was printed on separate substrates—Multi Substrate (MS) approach.

### 2.1. Single Substrate (SS) Approach

**Figure 4:**
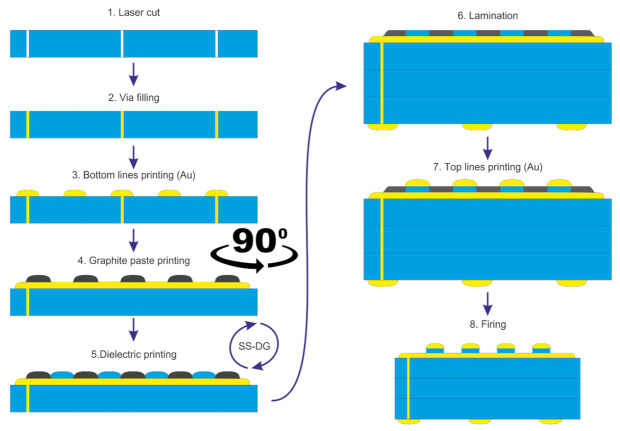
The manufacturing process using a screen-printed insulation layer.

In the first approach, all layers were screen printed on an LTCC substrate with vias connecting electrodes to the soldering pads on the bottom. The structure consisted of lower electrodes, then a screen-printed dielectric layer, and on the top perpendicular array of electrodes (Figure 4). To protect the bottom electrodes against contamination from the dielectric paste and to prevent shorts with the top electrode, a sacrificial layer of graphite was introduced. The 4440 (Electro-Science Laboratories Inc., King of Prussia, PA, USA) graphite paste was screen-printed to form strips perpendicular to the bottom layer and form a uniform surface together with the dielectric layer. Two manufacturing processes were tested: the sacrificial layer was printed either before the dielectric (SS-GD) or afterward (SS-DG). The dielectric layer should have low permittivity and similar mechanical properties and sintering behavior as the LTCC substrate. Therefore, a custom glass-ceramic thick film paste was prepared, using a 502K08 LTCC powder (Heraeus, Hanau, Germany), 10 wt.% of an organic binder—ethyl cellulose (Sigma Aldrich, Saint Louis, MO, USA), and the 9180 thinner (DuPont, USA). To ensure mechanical stability during printing and sintering of the top layer, the insulator needs to be sufficiently wider. In this case, a distance of 40 μm was used. Before printing the top electrodes, all ceramic layers were stacked together and laminated. This step was introduced in order to reduce the material waviness of the screen-printed layers. Such non-planarity would otherwise result in poor quality of the top layer. The lamination process was carried out under standard conditions recommended by the tape manufacturer—temperature of 70 °C, pressure 20 MPa for 5 min. Finally, the top conductive layer was screen-printed onto the prepared laminate.

### 2.2. Multi Substrate (MS) Approach

**Figure 5:**
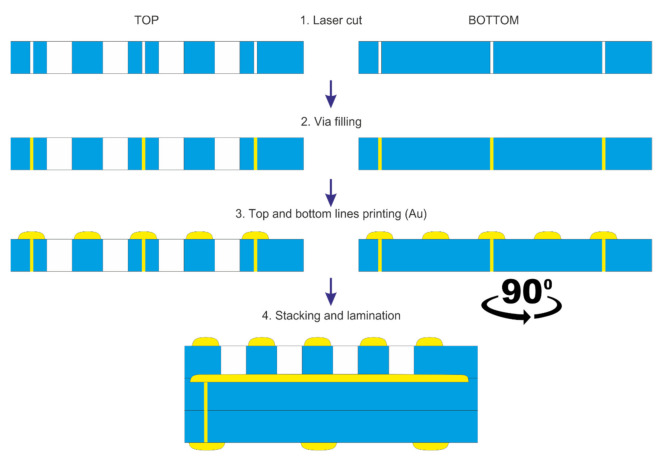
The manufacturing process using an LTCC insulation layer (Multi Substrate, MS).

The schematic diagram of the second process is presented in Figure 5. At first, the via holes were developed to connect the top electrodes with the rest of the system. They were filled with the same conductive paste as in the SS approach. Subsequently, the top stripe electrodes were screen-printed. Effectively, the bottom and the top electrodes were deposited on separate substrates. To expose the bottom electrodes, an array of openings, 150 μm × 200 μm each, was laser-cut in the dielectric layer (114 μm thick). Subsequently, the structure was stacked and laminated under standard conditions.

### 2.3. Sintering and Assembly

In both cases, the device was fired at the typical time-temperature profile with a 30-min hold at 875 °C in a box furnace LT 9/11 (Nabertherm, Lilienthal, Germany). In the case of the single substrate (SS) approach, the sample was additionally held for 1h at a temperature of 700 °C during firing to complete the removal of graphite from the surface. After sintering the LTCC module, an overglaze layer was applied on the bottom surface, using the QQ550 paste (DuPont) and fired at 550 °C/10 min hold in a belt furnace QA 41-6-54 (BTU, North Billerica, MA, USA. After firing, the ceramic part was cut into the desired shape. The complete LTCC readout board was soldered to an adapter printed circuit board (PCB), with OM-520 bismuth-silver-tin solder paste (Alpha Assembly Solutions, Somerset, NJ, USA). The adapter boards were equipped with Hirose FX10A-140P/14-SV connectors.

### 2.4. The GEM Detector Assembly

**Figure 6:**
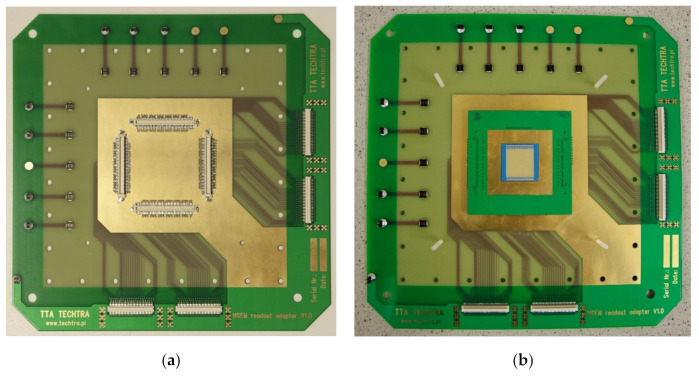
The PCB setup for ceramic readouts testing, (**a**) board with connectors; (**b**) board with connected LTCC readout module.

**Figure 7:**
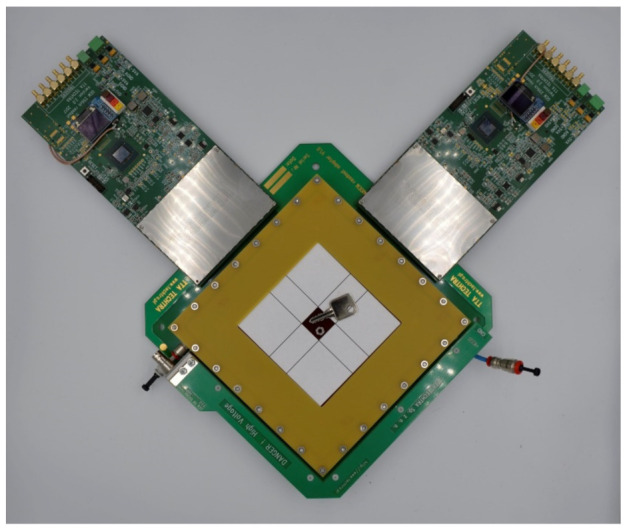
The Measurement setup—GEM detector with two Techtra 256ch electronic Readout V2.0 boards and metal samples placed on the active area of the detector.

Parallel to the development of the LTCC readout board, the test stand was prepared. To validate the readouts in an operational environment, the tests of a complete GEM detector were performed. For compliance, the framework of a standard CERN 10 × 10 cm^2^ detector was used [18,19]. GEM foils, drift electrode, spacers, gas-box frame, and cover with polyimide window. All of these parts were manufactured and assembled by Techtra Sp. Z o. o. The typical GEM readout boards serve two functions: electrical (collects the charge on readout strips) and mechanical (the board is a detector base and part of a gas box). The developed 2D LTCC readout modules are soldered on small adapter boards that need to be connected to a mounting PCB. For that purpose, the dedicated PCB was designed—the board is based on a standard CERN readout board, but instead of the readout strips, this board has connectors in the active area. The connectors merge the adapter board containing the LTCC readout module with a mounting PCB. The mounting PCB without (a), and with the LTCC readout module (b), is shown in Figure 6. This GEM detector base contains 4 pcs. of 130-pin Panasonic^®^ connectors and can be connected to any standard readout electronics designed for MPGD detectors. In our research, we used our own electronic system—the Techtra GEM 256ch Readout V2.0 [20]. The measurement setup includes a GEM detector with two 256ch electronic readout boards and metal samples placed on the active area of the detector are presented in Figure 7. The X-ray tube and radiation shield are not shown in the picture.

## 3. Results

Developed structures were thoroughly analyzed, initially through visual inspection and then characterized in the GEM detector by X-rays. Two samples were chosen for further analysis, one with the glass-ceramic dielectric layer (Single Substrate, SS) and one with the LTCC insulation layer (Multi Substrate, MS). The work described in this paper was the preliminary tests of the LTCC based readout boards for GEM detectors, so only one of each board type was measured. No statistical measurements on a larger readout boards group were performed.

### 3.1. Visual Inspection

**Figure 8:**
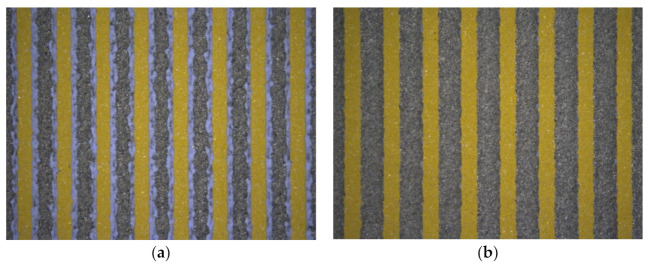
Analysis of the influence of graphite layer on electrode shape and surface quality, in dependence of printing sequence: (**a**) dielectric layer (blue) printed in between of graphite strips (SS-GD); (**b**) graphite layer (gray) printed after the dielectric layer (SS-DG).

The visual inspection was carried out using a DM4500 optical microscope (Leica, Germany), using 5× and 10× magnification with observation in the dark field to eliminate measurement error caused by light reflection. This step was carried out after each print in order to validate the processing path and detect faults. The most important task was to determine the influence of the graphite sacrificial layer on surface quality in the single substrate (SS) approach. Printing the top electrodes on an uneven surface due to the perpendicularly printed lower electrodes causes a decrease in resolution. Filling the gaps between the bottom strips with a graphite paste provides a flat surface and thus better screen-printing quality, while the sacrificial layer is burnt out during the firing process. Two screen printing sequences of the dielectric and graphite layer were tested; both are shown in Figure 8. Printing the graphite layer first (SS-GD) proved a better solution. Additionally, the graphite layer minimized the risk of short circuit occurrence and improved the visibility of the lower electrodes.

**Figure 9:**
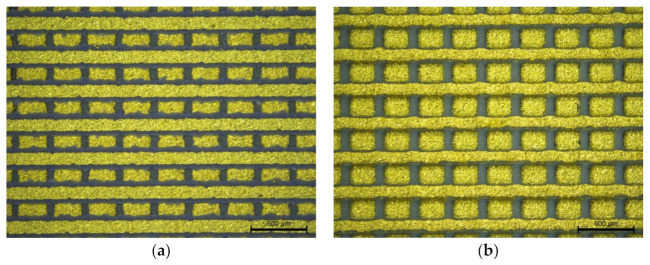
The image of the structure after co-firing utilizing two methods, (**a**) with a screen-printed dielectric layer (SS) and (**b**) with insulation made of LTCC tape (MS).

The comparison between the process with screen-printed dielectric on a single substrate (SS) and printing on multiple substrates (MS) was also performed visually. Results of the two manufacturing processes show that the fully screen-printed electrode array with the graphite sacrificial layer seems to provide better replication of the design and reproducibility of the upper electrode than screen printing on separate substrates. The shape of the deposited electrodes is close to the shape of the designed pattern. It is less susceptible to positioning errors during screen-printing and, as a result, contains fewer defects, primarily short circuits between electrode layers. Pictures of the readout area after co-firing are presented below in Figure 9.

**Figure 10:**
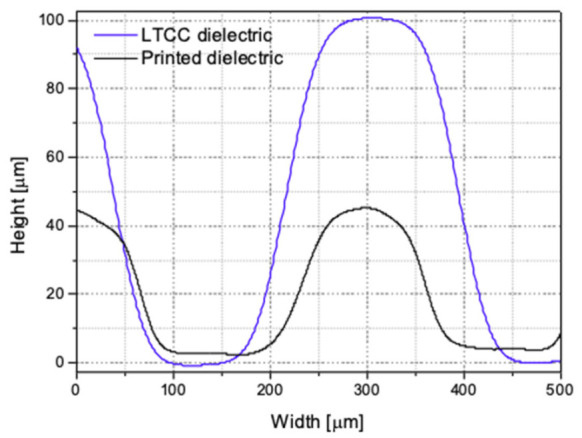
Surface topography profiles of LTCC readout boards manufactured with two methods.

The surface profiles of both structures are presented in Figure 10. They were obtained through software analysis (Leica Application Suite) of focal points on a series of images, taken using a small depth of field lens. As can be observed, the distance between the top and bottom electrodes is more than two times lower for the first method than for the second one. Therefore, the lower electrode is less shaded, and more electrons can reach there.

### 3.2. Analysis of LTCC Readout Boards Done with the Electrical and X-ray Methods

The electrical and X-ray characterization of three LTCC readout boards described in the previous section were conducted. Each board consists of 136 separate signals (68 X lines and 68 Y lines). The readouts can be electrically characterized by measuring: the shorts between lines, leakage current between lines, the conductivity of the lines from connector to end of a readout, the capacitance between lines, etc. Manual measurements or building a dedicated stand for automated tests is time-consuming. Alternatively, the operational parameters of the readout boards can be obtained by X-ray and noise measurements. Shorted lines (especially between the X and Y lines) significantly reduce the image quality of the radiographs, so the short-circuits number should be as small as possible. The shorts can be clearly visible in the channel noise level, so this method was chosen to investigate all readout boards concerning shorted lines. The detected shorts were additionally checked with resistance measurements. The results are presented in Table 1. These results show that the order of application of the dielectric and graphite layer makes a significant difference. The sacrificial layer is very important to prevent cross-pollution of the materials.

**Figure 11:**
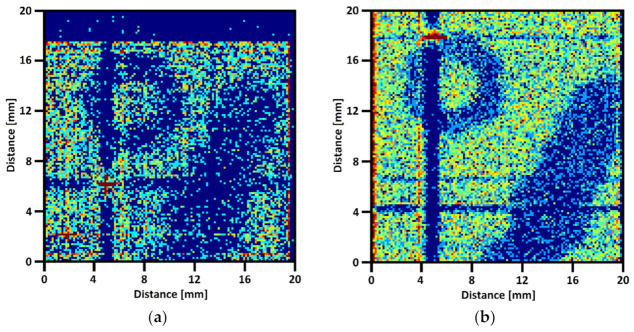
The radiographs of a key and an M6 nut (arrangement shown in Figure 7), measured on the (**a**) SS-GD; (**b**) MS readout board.

After the electrical measurements, the GEM detector was assembled using the LTCC readout boards, which were to be tested. The COOL-X pyroelectric X-ray source (Ametek, USA) was used during the tests. The energy of photons was mainly 8.05 keV (Cu) and 8.14 keV (Ta). The calculated detector gain for such photons was around 60,000. The standard Ar/CO_2_ (70:30) gas mixture for the MPGD was used. For the tests, a metal key and an M6 nut were placed on the detector and irradiated by the mentioned X-ray source for 3 h each. The radiographs obtained in these measurements are presented in Figure 11. The SS-DG board had too many short lines to reconstruct the radiograph properly.

The single shorted lines on other boards are clearly visible in the pictures as dark lines with red points at their intersection. Figure 11b looks better than Figure 11a because more events were detected. The measurement time and the X-ray lamp setup were the same for both experiments. Probably, the reason is that the dielectric used in the SS-GD method covers edges of the bottom lines (can be seen in Figure 9a), so some of the charges distributed on the bottom lines were smaller than the detection threshold. Using purpose-developed data processing algorithms, the resolution (pixel number) of the reconstructed images is two times greater than the number of XY electrodes. The algorithm calculates the event coordinates using the Gaussian approximation of the charges deposited on 10 neighboring lines—the center of a Gauss is the X or Y coordinate. The wide dark region on the top side of Figure 11a was caused by cracks in solder joints, while some of the strips were not connected with the electronic board. That was only the soldering defect, and it is not related to the technology of manufacturing readouts with a glass-ceramic insulation layer.

**Figure 12:**
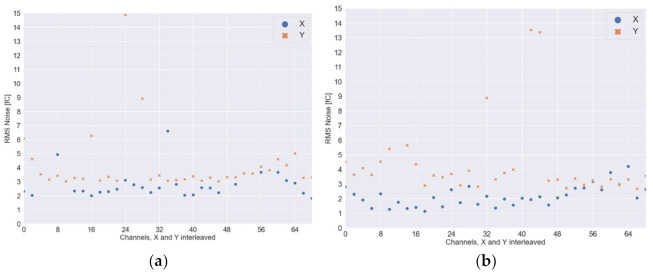
The RMS noise values for each channel of tested readout boards: (**a**) MS; (**b**) SS-GD readout board.

The data measured on the described detector was further processed to obtain the readout board’s performance. The electronic readout uses a switched-capacitor charge-to-digital converter. This type of ADC is sensitive to the capacitance of the input circuit—the noise level depends on readout strips capacitance. To equalize charge distribution between the top and bottom lines, the width of the top and bottom lines are different, where the pitch is the same. Hence, the distance between edges of adjacent strips on the top layer is larger than for the lines on the bottom side, and the capacitance and noise will also be different. In this way, the noise characteristics were calculated separately for the X and Y channels—the results are shown in Table 2. The RMS noise values for each channel are presented in Figure 12. Measurements were made on a fully assembled and working detector, with an ArCO_2_ gas mixture in a gas chamber and with high voltage enabled. We can see that the noise level on the Y-axis (bottom strips) is higher than on the X-axis. In addition, we can see that the noise levels are similar on MS and SS-GD boards.

The bottom electrodes of readout boards are further from the lowest GEM foil than the top strips, so the probability that electron reaches the bottom electrode is lower than that for the top electrode. The energy distribution of charges that reach a readout board was drawn on histograms separately for the X and Y lines. Then data containing signals from events were fitted with Gaussian function. The centers of the fitted curves represent the average value of the charges deposited on the selected lines. The percentage of charge deposition between the X and Y lines was calculated. Optimally, the charge should be equally distributed between X and Y lines. Unfortunately, the SS-GD was damaged during tests, and the charge distribution tests could not be performed. Table 3 presents calculated data for charge distribution on the developed multi-substrate (MS) readout board.

## 4. Discussion

The presented work shows that 2D readout boards for MPGD detectors with an active area of 20 × 20 mm^2^ can be successfully manufactured using LTCC technology. The readouts were tested in a working GEM detector—the technology was demonstrated in an operational environment. The two different methods were tested, and both readout boards can detect the X-rays. The fully screen-printed version of the detector, based on a single substrate (SS), can be simpler to manufacture, especially in high volumes. It can be adapted for other ceramic substrates, such as aluminum oxide (Al_2_O_3_) or nitride (AlN). Therefore, it was optimized for the best performance using a graphite sacrificial layer. However, it is the second method, which utilizes LTCC tape as a separator between the layers with electrodes (multi-substrate, MS), that gives better results with fewer defects and better uniformity. This confirms that LTCC-based readout boards are valid technology that should be further developed.

We suggest that the next stage of development should involve a larger readout board, developed using the MS method. The readouts with an active area of at least 70 × 70 mm^2^ could fit into the CubeSat module to investigate cosmic rays. Even larger boards could be manufactured for other applications; however, modular design would be a more flexible and cheaper solution. Several elements could be mounted very closely, without any frames around the active area, using BGA connections, to form a bigger panel. The second parameter that can be improved in further studies is the charge distribution uniformity between the top and bottom readout lines. Existing solutions achieve uniformity close to 50/50% by applying different widths of top and bottom strips for charge equalization. The same technique can be applied to ceramic-based readouts. 

Most importantly, the research shows that the LTCC-based GEM detectors are a viable solution worth pursuing. The performance and lifetime of a hermetically sealed GEM detector, used in space, for example, strongly depends on gas purity. Therefore utilization the materials with a very low outgassing ratio is critical for the application. The state-of-the-art GEM foils and readouts are made from polymer or epoxy-based materials, which outgassing ratio is higher than in metals or ceramics. The presented readout, in combination with already described LTCC GEM foils [15], can form a complete solution that is robust, precise, and can significantly reduce the outgassing problem. Therefore, further work will focus on the development and optimization of a fully ceramic solution. 

## Figures and Tables

**Table 1:** Shorts circuit traces in readout boards.

Board	Nr of Shorts	X Coordinates	Y Coordinates
SS-DG	27 (2 blocks)	23, 29, 47–49, 51, 53, 55, 58 60, 61, 63, 65–69	2, 4–10, 20, 24
SS-GD	2	36	47
		59	32
MS	1	22	58

**Table 2:** Noise characteristic of the detector with ceramic readout boards.

Board	Channels	Avg. Noise [fC]	Max Noise [fC]	RMS Noise [fC_RMS_]
MS	X (TOP)	7.87	17.85	2.26
	Y (BOT)	10.69	26.71	4.58
SS-GD	X (TOP)	7.77	18.25	2.86
	Y (BOT)	9.81	29.98	3.86

**Table 3:** Charge distribution between the top and bottom electrodes of the ceramic readout board.

Board	Channels	Gauss Center [pC]	Total Charge [%]
MS	X (TOP)	4.11	89.4
	Y (BOT)	0.49	10.6
